# Two Consecutive Runs of Veno-Venous Extracorporeal Membrane Oxygenation in a Peripartum Patient with COVID-19 Acute Respiratory Distress Syndrome

**DOI:** 10.1155/2021/2032197

**Published:** 2021-07-09

**Authors:** Nicolò Sella, Tommaso Pettenuzzo, Michele Della Paolera, Giulio Andreatta, Annalisa Boscolo, Alessandro De Cassai, Luisa Muraro, Arianna Peralta, Paolo Persona, Enrico Petranzan, Francesco Zarantonello, Eugenio Serra, Paolo Navalesi

**Affiliations:** ^1^Department of Medicine (DIMED), Padua University School of Medicine, Italy; ^2^Anesthesia and Intensive Care Unit, Padua University Hospital, Italy

## Abstract

Veno-venous extracorporeal membrane oxygenation (V-V ECMO) may be required to treat critically ill patients with COVID-19-associated severe acute respiratory distress syndrome (ARDS). We report the case of a 43-year-old peripartum patient, who underwent two sequential V-V ECMO runs. The first extracorporeal support was established for COVID-19 ARDS, as characterized by severe hypoxemia and hypercapnia (arterial partial pressure of oxygen to inspired oxygen fraction ratio 85 mmHg and arterial partial pressure of carbon dioxide 95 mmHg) and reduction of respiratory system static compliance to 25 mL/cmH_2_O, unresponsive to mechanical ventilation and prone positioning. After 22 days of lung rest, V-V ECMO was successfully removed and ventilator weaning initiated. A second V-V ECMO was required 7 days later, because of newly onset ARDS due to Pseudomonas aeruginosa ventilator-associated pneumonia. The second V-V ECMO run lasted 12 days. During both V-V ECMO runs, anticoagulation and ventilator settings were titrated through bedside thromboelastometry and electrical impedance tomography, respectively, without major complications. The patient was successfully decannulated, weaned from mechanical ventilation, and finally discharged home without oxygen therapy. At one-month follow-up, she showed good general conditions and no sign of respiratory failure.

## 1. Introduction

Severe acute respiratory syndrome coronavirus 2 (SARS-CoV-2) pandemic represents an enormous challenge for the healthcare system because of the imbalance between care demand and available resources for the treatment of critically ill patients with coronavirus disease 2019 (COVID-19) [1]. Whether extracorporeal membrane oxygenation (ECMO) should be considered for COVID-19 patients on a vast scale is still debated [2]. Indeed, despite being a high-demanding and resource-consuming technology, veno-venous (V-V) ECMO support proved to be beneficial in selected patients with severe COVID-19-associated acute respiratory distress syndrome (ARDS), resulting in a 62% in-hospital survival, quite similar to the rate observed in patients undergoing ECMO for non-COVID-19 ARDS [3].

We herein report the case of a peripartum patient, who underwent two consecutive V-V ECMO runs, the first for COVID-19 ARDS and the second for ARDS consequent to Pseudomonas aeruginosa infection.

## 2. Case Presentation

**Figure 1:**
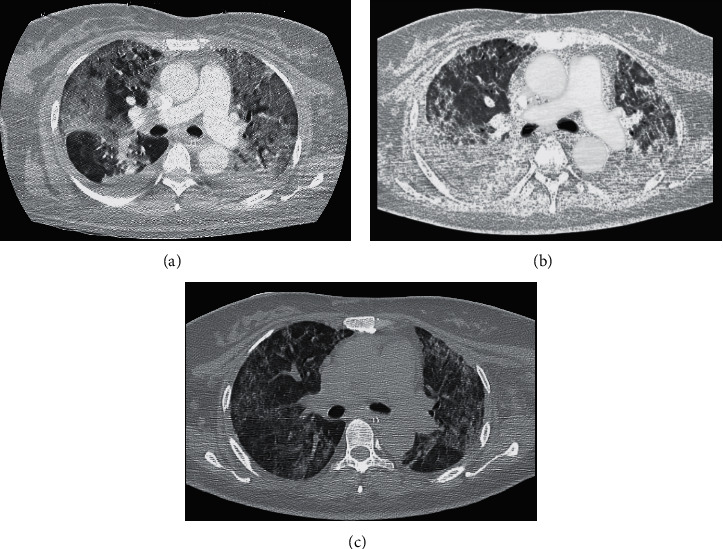
Axial chest computed tomography scan. (a) Before the first extracorporeal membrane oxygenation support. Diffuse bilateral ground glass opacity pattern and right pleural effusion are shown. (b) During the first extracorporeal membrane oxygenation support. Thickening of interstitial septa and diffuse crazy paving are shown. (c) After the first decannulation of extracorporeal membrane oxygenation. Lung aeration improved bilaterally with partial resolution of ground glass opacities.

A 43-year-old pregnant woman, with previous history of arterial hypertension, bronchial asthma, and mild obesity (body mass index 32), developed COVID-19 acute respiratory failure in her 34th gestational week, requiring hospital admission in northern Italy. She did not respond to noninvasive ventilation, intravenous dexamethasone, and SARS-CoV-2 convalescent plasma and was then transferred to the intensive care unit (ICU), where she was intubated, sedated, and mechanically ventilated in volume controlled mode with tidal volume (*V*_*T*_) 8 mL/kg of ideal body weight (IBW) and positive end-expiratory pressure (PEEP) titrated according to the low PEEP/inspired oxygen fraction (FiO_2_) table. Because of severe hypoxemia with arterial partial pressure of oxygen (PaO_2_) to FiO_2_ ratio (PaO_2_/FiO_2_) of 93 mmHg and cardiotocographic signs of fetal distress, an urgent cesarean section was performed on the same day, and a healthy baby was delivered without any COVID-19-related complications. After successful cesarean section, the patient received three cycles of prone positioning with PaO_2_/FiO_2_ consistently below 100 mmHg, while respiratory system static compliance (Crs) decreased to 24 mL/cmH_2_O. Chest computed tomography (CT) scan showed diffuse bilateral ground glass opacity pattern and right pleural effusion with no sign of pulmonary embolism (Figure 1(a)).

**Figure 2:**
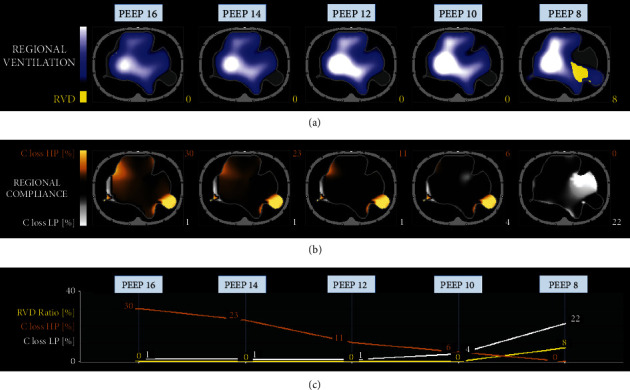
Electrical impedance tomography decremental positive end-expiratory pressure (PEEP) trial during the first extracorporeal membrane oxygenation support. In (a), the regional distribution of tidal ventilation at different PEEP values is shown by the blue–white gradient area, while the yellow area represents regional ventilation delay (RVD), whose percentage is indicated by the yellow number. In (b), regional compliance (C) is analyzed at different PEEP values. Compliance loss secondary to high PEEP (HP) is represented by the orange area (i.e., lung overdistension), while compliance loss due to low PEEP (LP) is represented by the white area (i.e., lung collapse). The percentage amounts of compliance loss associated with HP and LP are quantified by the orange and white numbers, respectively. In (c), RVD and compliance loss due to HP and LP at the different values of PEEP are plotted in order to facilitate the selection of the patient's best PEEP. This electrical impedance analysis shows the extreme heterogeneity of ventilation distribution, reflecting the severe and diffuse lung parenchyma abnormalities, documented also by chest computed tomography.

Despite paralysis, *V*_*T*_ reduction to 6 mL/kg IBW, and repeated recruitment maneuvers, respiratory system driving pressure was 19 cmH_2_O, with persisting refractory hypoxemia (PaO_2_/FiO_2_ 85 mmHg), hypercapnia (arterial partial pressure of carbon dioxide (PaCO_2_) 95 mmHg), and respiratory acidosis (pH 7.21). The patient was then transferred to our ICU in Padua, which is a referral center for extracorporeal life support^1^. On admission in our ICU, *V*_*T*_ was reduced to 4 mL/kg IBW, respiratory rate increased up to 30 breaths per minute, and PEEP titrated with electrical impedance tomography (EIT) [4]. Nevertheless, severe hypoxemia (PaO_2_/FiO_2_ 86 mmHg) and marked Crs reduction (25 mL/cmH_2_O) were confirmed, while maintaining respiratory system driving pressure within the lung protective range was unattainable. Therefore, 12 days after intubation, right femoral and internal jugular veins were cannulated, and V-V ECMO was initiated. Lung ultraprotective ventilation was established with *V*_*T*_ 2 mL/kg IBW and EIT-guided PEEP titration (Figure 2) [4, 5].

Anticoagulation therapy with enoxaparin was also initiated, targeting antifactor Xa activity between 0.3 and 0.7 IU/mL and monitoring whole blood coagulation by bedside thromboelastometry and platelet aggregometry [6]. Crs further deteriorated (8 mL/cmH_2_O), and a second chest CT scan revealed thickening of interstitial septa and diffuse crazy paving pattern, suggestive of pulmonary fibrosis (Figure 1(b)). Therefore, the patient received a cycle of systemic methylprednisolone. Percutaneous tracheostomy was performed while on V-V ECMO. After two cycles of prone positioning with poor response and 22 days of lung rest [5], Crs gradually improved, ventilation of the native lung was carefully increased, and extracorporeal support was finally successfully discontinued (Figure 1(c)). Six days after ECMO removal, the patient was weaned off the ventilator, and transfer to the referring ICU was planned.

**Figure 3:**
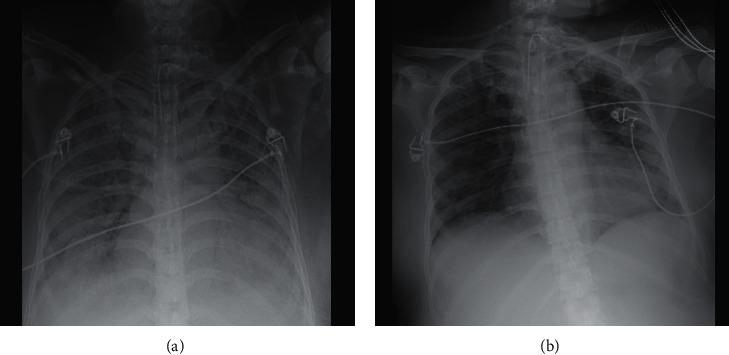
Chest X-ray. (a) A week after the first extracorporeal membrane oxygenation weaning. Bilateral pneumonia is shown. (b) After the second decannulation of extracorporeal membrane oxygenation. Partial resolution of bilateral pneumonia with persistence of diffuse loss of aeration is shown.

On day 7 after ECMO removal, however, the patient developed septic shock with leukocytosis (16.5 × 10^9^/L) and increased C-reactive protein and procalcitonin (450 mg/L and 1.52 mcg/L, respectively). High doses of norepinephrine infusion were required, and broad-spectrum antibiotic therapy with ceftazidime/avibactam, phosphomycin, linezolid, and caspofungin was initiated. New-onset ARDS was diagnosed (Figure 3(a)), as characterized by PaO_2_/FiO_2_ of 90 mmHg, PaCO_2_ of 65 mmHg, and pH 7.26. Deep sedation, paralysis, and volume controlled ventilation with *V*_*T*_ 5 mL/kg IBW and EIT-guided PEEP titration (16 cmH_2_O) were provided [4]. Prone positioning was attempted, but neither PaO_2_/FiO_2_ (89 mmHg) nor PaCO_2_ (66 mmHg) improved, while severe Crs reduction was confirmed (32 mL/cmH_2_O). Therefore, 8 days after ECMO removal, a left femoral-right jugular V-V ECMO was established, and lung ultraprotective ventilation was reinstituted. No problem was encountered during the second cannulation. We placed the draining cannula in the left femoral vein because the right femoral vein had already been utilized for positioning a central line. Microbiological cultures of bronchial aspirate samples tested positive for multidrug-resistant Pseudomonas aeruginosa, while real-time polymerase chain reaction for SARS-CoV-2 on nasopharyngeal swab tested negative. Patient's clinical conditions and chest X-ray slowly improved, and ECMO was discontinued 12 days later (Figure 3(b)). Ventilatory weaning was recommenced in neurally adjusted ventilatory assist mode, and after 4 days, the patient was transferred back to her ICU with minimal respiratory support. Two weeks later, she was discharged home without oxygen therapy. At the last follow-up, one month after hospital discharge, the patient was back home with her healthy baby, without need for oxygen therapy.

## 3. Discussion

We describe the case of a patient who was successfully supported with two consecutive V-V ECMO runs for different indications in the immediate postpartum period.

V-V ECMO has been previously applied as lifesaving rescue treatment for critically ill pregnant and postpartum patients. A retrospective analysis of the International Registry of Extracorporeal Life Support Organization between 1997 and 2017 found 280 peripartum patients who received ECMO for refractory cardiac or respiratory failure, with 70% overall maternal survival rate and no statistically significant difference in mortality between pulmonary versus cardiac indications or between V-V versus veno-arterial configuration [7].

However, data on the use of ECMO in COVID-19 peripartum women is limited. In a recent large international cohort study, including 1035 patients with COVID-19 who received ECMO support, 22 (2%) patients were pregnant, and 13 of them (1%) were cannulated with V-V ECMO because of COVID-19 ARDS [3]. Unfortunately, this study does not provide information on outcomes or complications [3]. A case series of 9 pregnant or peripartum patients supported with V-V ECMO for COVID-19 ARDS reported a median time of application of 10 days. All patients survived with two patients experiencing oxygenator thrombosis and one developing vaginal bleeding [8].

Anticoagulation management is challenging in peripartum COVID-19 ARDS, since multiple derangements of hemostasis may occur. Besides blood contact with the nonendothelial surface of the extracorporeal circuit activating coagulation, the physiologic pregnancy-related hemostatic changes and the hypercoagulable state typical of COVID-19 play a role [6]. In our case, we carefully kept ECMO blood flows ≥ 3.5 L/min to minimize the risk of clotting. Notably, no therapeutic intravenous anticoagulation was administered during ECMO. Instead, we chose an intermediate subcutaneous enoxaparin dosage (50 IU/kg b.i.d.) and titrated the anticoagulant therapy based on careful heparin activity surveillance and coagulation point-of-care testing. Worth remarking, we used heparin-coated ECMO circuits. This approach likely contributed to averting both thrombotic and hemorrhagic complications throughout V-V ECMO support.

Individualized mechanical ventilation settings and close monitoring of respiratory system mechanics were also adopted to prevent further ventilator-induced lung injury [4, 5]. In our experience, EIT proved to be a valuable tool for bedside evaluation of ventilation distribution and ventilation-perfusion matching, allowing patient-tailored PEEP titration and minimizing the risk of both lung overdistension and collapse [4, 9].

After recovery from the first COVID-19-related ARDS, our patient experienced severe ARDS secondary to bacterial infection. Patients with COVID-19 ARDS are particularly prone to develop ventilator-associated pneumonia (VAP), because of prolonged invasive mechanical ventilation, SARS-CoV-2-related lymphopenia, and corticosteroid or immunosuppressive therapy [10]. A recent retrospective cohort study, including 50 consecutive patients requiring ECMO for COVID-19 ARDS, showed a very high incidence of late-onset VAP (86%) and frequent VAP recurrence (79%), despite adequate antimicrobial treatment, mainly caused by inducible AmpC-cephalosporinase-producing Enterobacteriaceae and Pseudomonas aeruginosa [10]. Interestingly, a higher incidence of VAP in COVID-19 patients was observed, compared with a historical cohort of patients supported with ECMO for severe influenza-associated ARDS, whereas extubation rate and mortality did not differ [10].

Two nearly consecutive V-V ECMO runs are uncommon in ARDS patients and are generally caused by the recurrence of the primary disease [8]. Among peripartum women with COVID-19 ARDS, Barrantes et al. reported a single case necessitating postpartum V-V ECMO recannulation consequent to resumption of acute respiratory failure [8]. Despite being successfully weaned also from the second ECMO run, the authors could not provide data on the patient outcome after hospital discharge, because she was still in hospital at the time of case reporting.

To the best of our knowledge, this is the first report of a peripartum patient supported with two consecutive V-V ECMO runs for different indications, who was successfully discharged at home, suggesting that a second V-V ECMO in COVID-19 patients is not contraindicated for severe ARDS of new onset, unrelated to the initial disease.

## Data Availability

The data that support the findings of this study are available from the corresponding author upon request and have not been previously published.

## References

[1] Tonetti T., Grasselli G., Zanella A. (2020). Use of critical care resources during the first 2 weeks (February 24-March 8, 2020) of the COVID-19 outbreak in Italy. *Annals of Intensive Care*.

[2] Shekar K., Badulak J., Peek G. (2020). Extracorporeal Life Support Organization Coronavirus Disease 2019 interim guidelines: a consensus document from an international group of interdisciplinary extracorporeal membrane oxygenation providers. *ASAIO Journal*.

[3] Barbaro R. P., MacLaren G., Boonstra P. S. (2020). Extracorporeal membrane oxygenation support in COVID-19: an international cohort study of the Extracorporeal Life Support Organization registry. *Lancet*.

[4] Sella N., Zarantonello F., Andreatta G., Gagliardi V., Boscolo A., Navalesi P. (2020). Positive end-expiratory pressure titration in COVID-19 acute respiratory failure: electrical impedance tomography vs. PEEP/FiO2 tables. *Critical Care*.

[5] Schmidt M., Pham T., Arcadipane A. (2019). Mechanical ventilation management during extracorporeal membrane oxygenation for acute respiratory distress syndrome. An international multicenter prospective cohort. *American Journal of Respiratory and Critical Care Medicine*.

[6] Boscolo A., Spiezia L., Correale C. (2020). Different hypercoagulable profiles in patients with COVID-19 admitted to the internal medicine ward and the intensive care unit. *Thrombosis and Haemostasis*.

[7] Ramanathan K., Tan C. S., Rycus P. (2020). Extracorporeal membrane oxygenation in pregnancy: an analysis of the extracorporeal life support organization registry. *Critical Care Medicine*.

[8] Barrantes J. H., Ortoleva J., O'Neil E. R. (2021). Successful treatment of pregnant and postpartum women with severe COVID-19 associated acute respiratory distress syndrome with extracorporeal membrane oxygenation. *ASAIO Journal*.

[9] Zarantonello F., Andreatta G., Sella N., Navalesi P. (2020). Prone position and lung ventilation and perfusion matching in acute respiratory failure due to COVID-19. *American Journal of Respiratory and Critical Care Medicine*.

[10] Luyt C. E., Sahnoun T., Gautier M. (2020). Ventilator-associated pneumonia in patients with SARS-CoV-2-associated acute respiratory distress syndrome requiring ECMO: a retrospective cohort study. *Annals of Intensive Care*.

